# The Influence of Guiding Concept on the Accuracy of Static Computer-Assisted Implant Surgery in Partially Edentulous Cases: An In Vitro Study

**DOI:** 10.3390/medicina61040617

**Published:** 2025-03-28

**Authors:** David Kasradze, Ričardas Kubilius

**Affiliations:** Department of Maxillofacial Surgery, Lithuanian University of Health Sciences, 50161 Kaunas, Lithuania; ricardas.kubilius@lsmu.lt

**Keywords:** static computer-assisted implant surgery, guided implant placement, implantology, guiding system, partial edentulism

## Abstract

*Background and Objectives*: Static Computer-Assisted Implant Surgery (sCAIS) can be performed with different drill guiding systems. This study aimed to compare the accuracy of two guiding concepts of sCAIS in partially edentulous cases. *Materials and Methods*: Forty polyamide models of partially edentulous maxillae with seven implantation sites were fabricated. In total, 140 replica implants were placed with keyless (KL) and drill-key (DK) guiding systems using static, full-arch, tooth-supported surgical guides. Three-dimensional crestal and apical, angular and vertical deviations from the planned implant positions were compared using Mann–Whitney U and Kruskal–Wallis H tests. Intergroup homogeneity of variance homogeneity was examined using Levene’s test to assess the precision. *Results*: Overall median 3D crestal and apical deviations of implants placed in the KL group were significantly higher compared to the DK group (0.86 mm [0.63–0.98] vs. 0.72 mm [0.52–0.89], *p* = 0.006 and 1.26 [0.98–1.52] vs. 1.13 [0.70–1.45], *p* = 0.012). In the subgroup analysis, implants placed with a KL system showed higher 3D crestal (*p* = 0.029), 3D apical (*p* < 0.001) and angular (*p* < 0.001) deviations in the extended anterior area, higher 3D crestal (*p* < 0.001) deviations in the proximal posterior single-tooth gap and higher vertical (*p* < 0.001) deviations in the distal site of free-end situation. Contrarily, the KL group showed lower 3D crestal (*p* = 0.007), 3D apical (*p* < 0.001), angular (*p* < 0.001) and vertical (*p* = 0.003) deviations in the distal posterior single-tooth gap, lower 3D apical (*p* = 0.007) and angular (*p* = 0.007) deviations in the distal site of free-end situation and lower vertical (*p* = 0.019) deviations in the proximal site of free-end situation. *Conclusions*: The deviations of both guiding concepts did not exceed the recommended safety margins. Statistically significant differences in deviations were found between two guiding concepts. Guiding concepts with superior accuracy varied across different sites of implantation.

## 1. Introduction

With the introduction of three-dimensional dental software in the late 1990s, the digitization of implant dentistry has been trending ever since [1]. It has impacted virtually every subject in the field of dentistry—from manufacturer and dental professional to patient and scholar. As for implant dentistry, digital advancements and computer-assisted surgery were directed to prompt its primary objectives—achieve predictable long-term treatment outcomes, reduce the risk of complications and improve patients’ experience [2]. This led to increased interest in the prosthetically-driven approach [1,3], especially with digital technologies becoming more affordable.

Computer-assisted implant surgery can be categorized into static and dynamic systems. Dynamic computer-assisted implant surgery utilizes preoperative CBCT images and intraoperative optical tracking to provide real-time feedback and drill navigation. In contrast, static computer-assisted implant surgery (sCAIS) relies on prefabricated surgical guides with predefined guide holes for static navigation. While sCAIS requires additional costs and time for treatment planning, it reduces direct working time [4,5,6], allows for more accurate transfer of the pre-planned implant position to the clinical environment than freehand placement [7,8,9,10], and helps avoid tissue augmentation [11,12]. The correct implant position is crucial for long-term success, as optimal implant-to-tooth, inter-implant distance, depth, and angulation contribute to proper occlusion, restoration design, balanced implant loading, and the stability of peri-implant tissues [13,14,15].

Despite these advantages, it is premature to consider sCAIS as universally advantageous in all cases. Firstly, the use of static guides can be limited by physiological factors such as mouth opening or the fact that they can break [16,17]. Secondly, deviations from the planned implant positions can compromise treatment or lead to complications related to implant positioning. Discrepancies between planned and actual implant positions are the result of cumulative errors occurring at different stages of the sCAIS workflow [18]. Therefore, identifying potential sources of error requires standardized study designs. Earlier studies have indicated that differences in guiding concepts and guide design could affect the accuracy of sCAIS [19,20,21,22,23,24,25,26].

The guiding concepts used in sCAIS can be categorized into drill-key and keyless systems. Drill-key (DK) systems utilize drill-specific keys mounted to the handle or the drills, which fit the sleeve hole in the surgical guide. Varying drill-key heights enable adjustment of the drill guiding distance and depth of implant placement [24]. Keyless (KL) systems achieve drill-to-sleeve fitting through a modified drill shank that fits the sleeve hole. Keyless guiding systems were designed to reduce the number of surgical components and tolerance between them to enhance the accuracy [23]. However, due to the relative recency of KL systems, there is a lack of standardized studies on the influence of guiding concepts on accuracy [24]. The results of recently published studies on the impact of guiding concepts on accuracy of sCAIS in partially edentulous cases are inconsistent and studies included limited variety of partial edentulism types [22,23,24,25].

Therefore, this in vitro study aimed to compare the accuracy of two guiding concepts of sCAIS in partially edentulous scenarios. The null hypothesis was that the guiding concept would have no effect on the accuracy of sCAIS across different types of partial edentulism.

## 2. Materials and Methods

**Figure 1:**
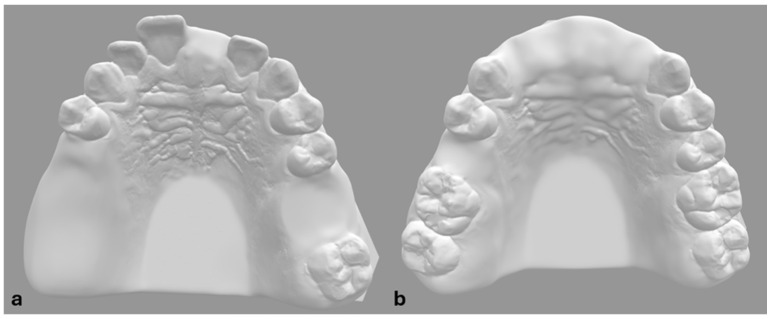
Digital prototypes of maxillary models with designed edentulous areas. Prototype model (**a**) with edentulous sites at FDI 15, 16, 17, 21, and 26. Prototype model (**b**) with edentulous sites at FDI 11, 12, 15, 21, and 22.

**Figure 2:**
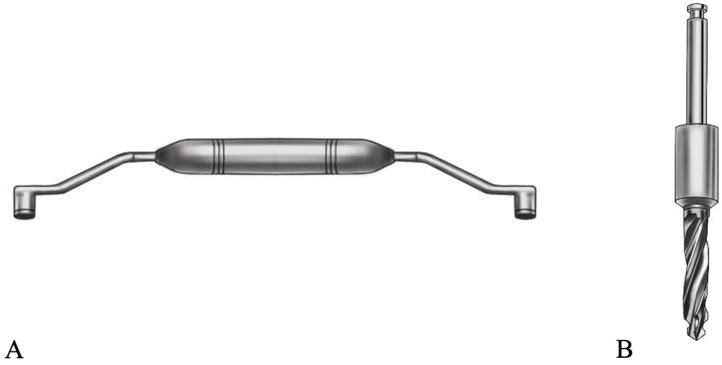
Figure illustrating a drill-handle used in DK system (**A**) and a drill with modified shank used in KL system (**B**).

**Figure 3:**
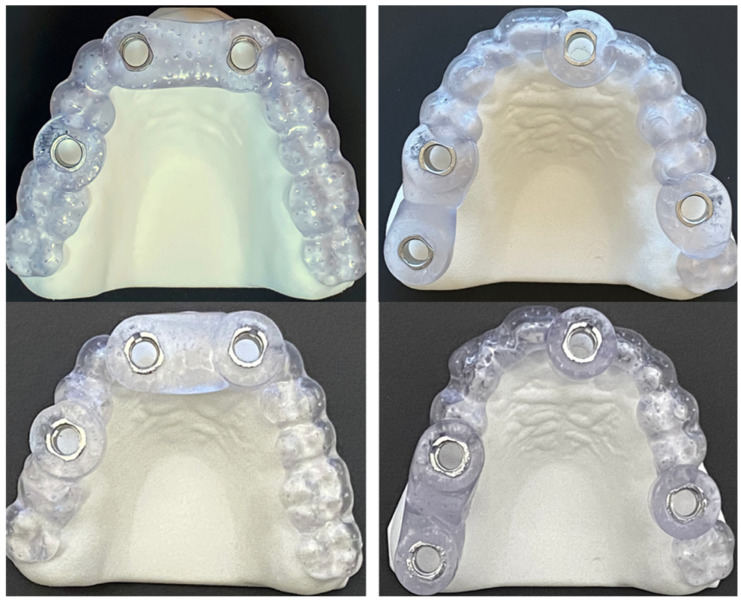
Picture illustrating full-arch supported guides for drill-key (DK) system in upper row, and keyless (KL) system in lower row.

De-anonymized CBCT and optical scan data of fully dentulous maxilla were used to create two prototype models of partially edentulous maxillae (Figure 1). To simulate different types of partial edentulism, teeth at the Federal Dentaire Internationale (FDI) positions 15, 16, 17, 21 and 26 were digitally removed in model A and at FDI positions 11, 12, 21, 22 and 15 in model B (Figure 1). Implantation sites were designed as fully healed alveolar sockets with sufficient alveolar ridge for implant placement. Forty polyamide-12 (PA2200) dental models were then fabricated with EOS P 396 printer using Selective Laser Sintering (SLS) technology. Cone bean computed tomographies (80 µm voxel size, Orthoplos SL 3D, Dentsply Sirona, Charlotte, NC, USA) and optical surface scans (Medit i700, Medit, Seoul, Republic of Korea) of each prototype model were acquired. Digital datasets were uploaded to the digital treatment planning software (exoplan Rijeka 3.1, Exocad, Darmstadt, Germany) to perform digital wax-up and implant positioning to designated implantation sites. To evaluate the influence of guiding concepts, two different implant systems were selected—Straumann BLT (Straumann, Basel, Switzerland) for DK group and Megagen Anyridge (Megagen, Daegu, Republic of Korea) for KL group (Figure 2). Dimensions of selected implants were 4.1 × 10 mm for Straumann BLT and 4 × 10 mm for Megagen Anyrdige. Two full-arch, tooth-supported surgical guides for each implant system were designed by the same investigator (Figure 3). For the KL group, the free drilling distance (FDD), defined as the distance from the bottom of the sleeve to the tip of osteotomy [26], was 17 mm, while for the DK group, it was 14 mm. Prior to selection, several different radial sleeve offsets were tested by an experienced investigator in surgical guide manufacturing to ensure the most adequate fit, and was set to 0.025 mm. The surgical guide bottom offset was set to 0.1 mm, and material thickness to 3 mm. A total of 40 guides, one for each dental model, were fabricated using digital light processing (DLP) with the Asiga Max UV 3D printer (Asiga, Sydney, Australia) and compatible printing material for surgical guides (DentaGuide, Asiga). After 3D printing, passive fit of the guides was checked on the models. The original manufacturer’s metal sleeves were incorporated into the corresponding sleeve holes and verified by a dental technician experienced in surgical guide manufacturing. The drilling sequence was carried out according to the manufacturer’s protocol using new drill sets.

### 2.1. Study Groups

Two guiding concepts (DK and KL) were compared in seven implantation sites of partially edentulous models. Full arch tooth supported surgical guides were used to place implants at FDI 15, 17, 21 and 26 sites in model A and at FDI 12, 22 and 15 sites in model B. Implantation sites were simulating six subgroups of edentulous areas:

- Anterior single-tooth gap (AntSTG) at FDI 21 site (*n* = 10 implants);
- Anterior extended edentulous area (AntExt) at FDI 12 and 22 sites (*n* = 20 implants);
- Proximal posterior single-tooth gap (ProPosSTG) at FDI 15 site (*n* = 10 implants);
- Distal posterior single-tooth gap (DisPosSTG) at FDI 26 site (*n* = 10 implants);
- Proximal site of distal extension area (ProDE) at FDI 15 site (*n* = 10 implants);
- Distal site of distal extension area (DisDE) at FDI 17 site (*n* = 10 implants).

### 2.2. Accuracy Measurements

**Figure 4:**
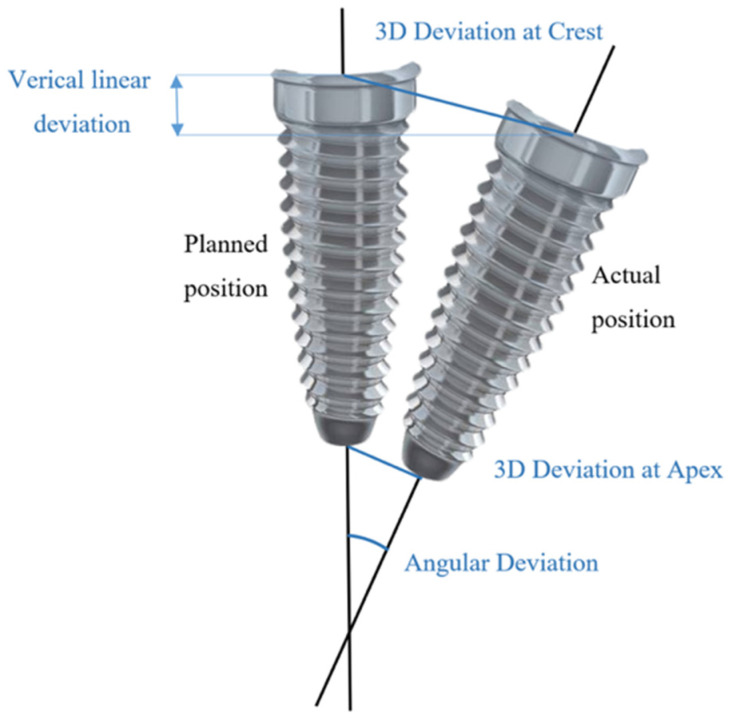
Schematic definition of measured deviations between planned and actual implant positions.

After implant placement, scan bodies were screwed onto the implants with respect to manufacturer’s recommendations and digital dental impression data were acquired (Medit i700, Medit). Standard Tesselation Language (STL) datasets of planned and post-op implant positions were uploaded to the 3D inspection software (Zeiss Inspect Optical 3D, Carl Zeiss, Oberkochen, Germany). Each dataset with actual implant positions was superimposed with a reference model using teeth occlusal surfaces as a reference. To evaluate the deviations between planned and actual implant positions, 3D apical and crestal, vertical linear and angular distances were measured (Figure 4). To conduct measurements, best-fit cones and planes on coronal and apical surfaces for virtual implants were generated. Central axes for each cone were generated automatically by the software. Intersection points between central axes and apical and coronal planes were referred to as crestal and apical points of virtual implants. Euclidean distances between corresponding intersection points of nominal and actual implants were considered as 3D apical and crestal deviations. Vertical linear deviation was considered the perpendicular distance from the crestal central point of the actual coronal plane to the nominal plane. Angular distance was a measure of angle between the central axes of nominal and actual virtual implants. All digital datasets are archived and available upon reasonable request.

The accuracy of implants was compared between study groups. The International Organization for Standardization (ISO) defines accuracy by trueness and precision [27]. Trueness refers to the closeness of agreement between the test results and a reference value which was defined by deviations between planned and actual implant positions. Precision refers to the closeness of agreement between the test results and is defined by a variability between the measurements and was expressed as standard deviations of the results, whereas less precision was reflected by a larger standard deviation.

### 2.3. Statistical Analysis

Sample size was calculated based on previous data by Sittikornpaiboon et al. [24], who reported results on similar guiding concepts. A global 3D crestal deviation was reported to be 0.56 ± 0.19 mm for the drill-key guiding concept and 1.09 ± 0.12 mm for the keyless guiding concept. Calculation using Noether’s method suggested a minimum of *n* = 9 per study group by setting of two-sided α = 0.05 and power = 0.95.

Statistical analysis was conducted by experienced investigator with IBM SPSS Statistics software 29.0 (SPSS Inc., Chicago, IL, USA). First, the descriptive statistics were summarized including mean, standard deviation, median, minimum and maximum values, 25th and 75th percentiles. The normality of the data distribution was evaluated using histograms, Q–Q plots, and the Shapiro–Wilk and Kolmogorov–Smirnov tests. Intergroup homogeneity of variance homogeneity was examined using a Levene’s test. The Kolmogorov–Smirnov test was performed on the general sample and showed that the distribution of angular and vertical deviations departed significantly from normality (D (140) = 0.090, *p* = 0.008 and D (140) = 0.075, *p* = 0.05). Since the sample size data of separate subgroups of implantation sites was small, a Shapiro–Wilk test was performed and showed significant departure from normality of distribution measurements in part of the subgroups. Based on these outcomes and visual examination of the histograms and the Q–Q plots, it was decided to use a non-parametric test. The Mann–Whitney U test was used to compare distributions of deviations between the two groups on overall data and on separate datasets of different implantation sites. The Kruskal–Wallis H test was used to determine if there are statistically significant differences between the six implantation sites. *p* values of <0.05 were considered as statistically significant. Pairwise comparisons were performed using Dunn’s procedure with a Bonferroni correction for multiple comparisons with statistical significance accepted at the *p* < 0.003 level (*p* < 0.05/15). The methodology was reviewed by an independent statistician. The article is reported according to Checklist for Reporting In-vitro Studies (CRIS) guidelines [28].

## 3. Results

A total of 560 measurements were obtained after registration of four deviation parameters for each of the 140 placed implants, all of which were included in the study. Seventy Straumann BLT 4.1 × 10 mm implants were placed with the DK guiding system and seventy Megagen Anyrdige 4 × 10 mm implants with the KL guiding system. Ten implants per system were placed in AntSTG, ProPosSTG, DisPosSTG, ProDE and DisDE implantation sites, whereas AntExt area included two implantation sites resulting in twenty implants per system. No statistically significant differences in medians of deviations and homogeneities of variances between the two implantation sites of AntExt area (FDI 12 and 22) were observed.

Overall mean 3D deviations for the KL system were 0.84 ± 0.26 mm at implant crest, 1.28 ± 0.41 mm at apex, angular—3.37 ± 1.43°, and vertical linear—0.46 ± 0.29 mm. For the DK system, the mean 3D deviation at implant crest was 0.71 ± 0.26 mm, at apex—1.08 ± 0.45 mm, angular—3.06 ± 1.72°, and vertical linear deviation—0.45 ± 0.33 mm. The differences in median 3D crestal and apical deviations between the two groups were statistically significant with values of *p* = 0.006 and *p* = 0.012, respectively. In terms of precision, the Levene’s test for each deviation measurement showed no statistically significant differences, suggesting the equal variances of the data between the two groups. Descriptive statistics are summarized in Table 1.

### Different Sites of Implantation

**Figure 5:**
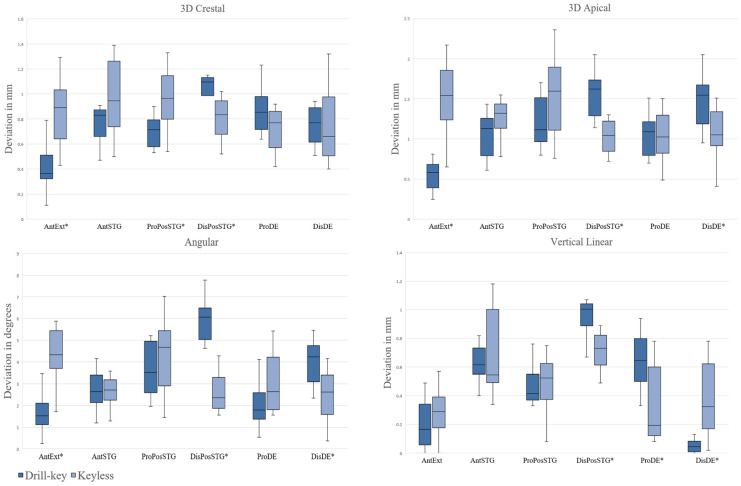
Box plots demonstrating 3D crestal, 3D apical, angular and vertical linear deviations when drill-key or keyless guiding systems were used in each of the implantation sites. Significant differences by Mann Whitney U test denoted as * for *p* < 0.05. AntExt—anterior extended edentulous area, AntSTG—anterior single tooth gap, ProPosSTG—proximal posterior single tooth gap, DisPosSTG—distal posterior single tooth gap, ProDE—proximal site of distal extension, DisDE—distal site of distal extension. Drill key—Straumann BLT 4.1 × 10, Keyless—Megagen Anyridge 4 × 10.

A separate analysis was conducted by comparing the deviation distributions of two guiding concepts in particular sites of implantation. In an AntSTG implantation site, no statistically significant differences between the groups of guiding concepts were observed. In an AntExt area, higher 3D crestal, apical and angular deviations of implants were observed when placed with the KL system compared to the DK system (0.89 mm [0.64–1.03], 1.54 mm [1.24–1.86], 4.33° [3.70–5.44] vs. 0.37 mm [0.32–0.51], 0.58 mm [0.39–0.68], 1.52° [1.12–2.11] with *p* = 0.029, *p* < 0.001 and *p* < 0.001, respectively). In the ProPosSTG site, implants in the KL group exhibited statistically significant higher 3D crestal deviations compared to the DK group (0.97 mm [0.80–1.15] vs. 0.72 mm [0.58–0.79] with *p* < 0.001). Contrarily, in the DisPosSTG site, implants in KL group showed lower 3D crestal, apical, angular and vertical deviations when compared to the DK group (0.84 mm [0.68–0.95], 1.05 mm [0.85–1.22], 2.36° [1.88–3.30], and 0.73 mm [0.61–0.82] vs. 1.10 mm [0.99–1.13], 1.62 mm [1.29–1.74], 6.06° [5.03–6.49], and 1.01 mm [0.89–1.04] with *p* = 0.007, *p* < 0.001, *p* < 0.001, and *p* = 0.003, respectively). Similarly, higher vertical deviations in the ProDE site and higher 3D apical and angular deviations in the DisDE site of implants placed with the DK system compared to the KL system were observed (0.65 mm [0.50–0.80] vs. 0.20 mm [0.12–0.60] with *p* = 0.019 and 1.55 mm [1.19–1.67], 4.23° [3.09–4.75] vs. 1.05 mm [0.92–1.34], 2.63° [1.59–3.41] with *p* = 0.007, *p* = 0.007, respectively). Vertical linear deviations in the DisDE site were statistically significantly higher for the KL group compared to the DK group (0.33 mm [0.17–0.62] vs. 0.05 mm [0.01–0.08] with *p* < 0.001). The box plots of deviations of the two guiding concepts across the sites of implantation are demonstrated in Figure 5.

Additionally, a Kruskal–Wallis H test was run to determine if there were significant differences in deviations between six subgroups of implantation locations. In the DK group, distributions of deviations were not similar for all subgroups, as assessed by visual inspection of the boxplots. The distributions of 3D crestal, 3D apical, angular and vertical deviations were statistically significantly different between the subgroups of implantation sites (*p* < 0.001). In the KL group, distributions of deviations were similar for all groups, as assessed by visual inspection of the boxplot. Median 3D apical, angular and vertical deviations were statistically significantly different between groups (*p* = 0.002, *p* < 0.001 and *p* < 0.001, respectively). The post hoc pairwise analysis using Dunn’s procedure with Bonferroni correction for multiple comparisons revealed statistically significant differences in median deviations between subgroups of implantation sites in both groups (Table S1).

## 4. Discussion

The present study examined the influence of guiding concepts on the accuracy of sCAIS in partially edentulous dental models. To the best of our knowledge this is the first in vitro study to investigate the impact of a guiding system in this variety of implantation sites. Study models included anterior extended area, anterior single tooth gap, two posterior single tooth gaps and two sites in distal extension area. Mean implant deviations in the present study were clinically acceptable. They did not exceed the 2 mm safety margins that are recommended with respect to clinico-anatomical reasons by separate authors [3,29,30,31].

The null hypothesis was rejected due to statistically significant differences in 3D crestal and apical deviations between the two groups. Higher trueness values of implants placed with the DK system compared to the KL system were observed in overall data. These findings are in agreement with results of earlier in vitro studies comparing similar guiding concepts by Guentsch et al. [22] and Sittikornpaiboon et al. [24]. Differences in free drilling distance and drill guidance length could be the possible factors to impact the results. Earlier studies have suggested that higher FDD correlated with increased 3D crestal, apical and angular deviations [26,32]. In the present study, the FDD varied in the study groups, from 14 mm in the DK group to 17 mm in the KL group. In addition, previous authors have suggested to increase the guiding length of the drill by longer sleeves or higher drill-keys to reduce deviations of sCAIS [19,26]. However, these parameters could be modified only with the DK system by choosing the bone-to-sleeve distance and different drill-keys, whereas the KL system offers a fixed height offset of the sleeve.

The findings of previous studies comparing DK and KL guiding concepts are inconsistent. By comparison, in a recent systematic review and meta-analysis authors reported higher 3D crestal deviations of implants placed with DK systems when compared to KL systems in partially edentulous cases [25]. Authors suggested that KL systems have superior drill deviation control, due to closer fit of surgical components and reduced tolerance to lateral movements. Keyless guiding systems achieve a drill-to-sleeve fitting with less surgical guide components than drill-key systems. A modified drill shank fits the sleeve hole and, therefore, the need for drill-keys is eliminated. The tolerance between surgical guide components, such as sleeve hole, sleeve, drill-key and drill, was shown to have negative impact on the accuracy of sCAIS [19,20,21,33,34]. Furthermore, authors have suggested that surgical guides without metal sleeves exhibited higher accuracy due to the lower number of surgical components and the tolerance gaps between them [23,35,36]. Inconsistent results among the studies are partially mirrored in the present study, as the guiding concept with higher accuracy varied across different sites of implantation. Significantly lower deviations of implants placed with the KL system compared to the DK system were observed in the distal extension site and the distal posterior single-tooth gap, but higher in anterior sites of implantation.

The possibility, of sCAIS being site-sensitive has been suggested in a study by el Kholy et al. [37]. The authors found that the accuracy of implants placed in the sites with bilateral tooth support was higher compared to the implants in the distal extension sites. Previous authors have suggested the use of guide stabilizers and optimal guide material in distal extension sites to address the impact of guide tilting and bending on the accuracy of sCAIS [28,38,39]. In the present study, a post hoc pairwise analysis revealed significant differences in deviations between implantation sites in both groups (AntExt-AntSTG, AntExt-DisPosSTG, AntExt-ProDE, AntExt-DisDE, AntSTG-DisPosSTG, AntSTG-DisDE, DisPosSTG-ProDE, ProPosSTG-DisDE, DisPosSTG-DisDE, ProDE-DisDE), suggesting that the results on the accuracy of sCAIS in this particular site of implantation should not be generalized across all types of partial edentulism. Different sites of implantation are characterized by different distribution and morphology of surrounding teeth that could influence the accuracy of sCAIS. The influence of the implantation site was different among two study groups. Higher angular deviations of implants placed with the DK system in the DisDE site when compared to the AntExt area were observed, whereas the results in the KL group were opposite with significantly lower 3D crestal, apical and angular deviations in the DisDE site. Our findings suggest that site of implantation impacts the accuracy of guiding systems differently. However, the present study was primarily designed to evaluate the influence of the guiding concepts on the accuracy of sCAIS; therefore, the role of the implantation site should be investigated in future studies.

The experimental design of the present study allowed the standardization of a number of possible confounding factors, such as physical properties and design of the guide, material density, number, morphology and distribution of supporting teeth, morphology of the alveolar ridge, data acquisition and measurement equipment. However, the interpretation of the results should consider the study’s limitations. Firstly, 3D printed polyamide models were used, which in our experience felt harder than cortical bone and required greater pressure on pilot drills. Secondly, the free drilling distance varied between the study groups. Thirdly, the macroscopic design of the two implant systems was different. Both implants had a tapered shape but the implants in the KL group featured more aggressive threads than in the DK group. Previous studies have shown that the macroscopic aggressiveness of the implants could influence the accuracy [34,40,41]. Fourth, metal sleeves were used for both implant systems to standardize guide designs. Part of the previous studies suggests that a sleeveless design leads to superior accuracy of the sCAIS [34,36]. Fifth, the physical properties of the guide material could affect the outcomes, especially in distal free-end situations [28]. Finally, the in vitro design omitted factors related to a clinical setting that might affect the accuracy of the sCAIS, such as patient movements, limited mouth opening, presence of saliva and blood and compromised accessibility or visibility [31,42]. In addition, the dental models did not simulate soft tissues, which in a clinical environment could affect the fitting and stabilization of the surgical guide [43]. In a meta-analysis by Bover-Ramos et al., it was concluded that in vitro studies reported higher accuracy results when compared to cadaver or clinical studies [31]. Therefore, the accuracy results of in vitro studies should not be translated to clinical situations. Nevertheless, the findings can offer insights on the influence of technical variables, which are challenging to compare in non-isolated study conditions. Further in vitro studies should focus on a high level of standardization of study designs. To externally validate the results, future clinical studies are needed to evaluate the influence of guiding concepts in different types of partial edentulism.

## 5. Conclusions

The deviations of both guiding concepts did not exceed the recommended safety margins. Significant differences were found between guiding systems in different sites of implantation. The DK system showed significantly higher crestal, apical, and angular accuracy in the anterior extended area and proximal posterior STG, as opposed to distal extensions and distal posterior STG, where the KL system exhibited superior results. The accuracy results of in vitro studies are not applicable to clinical settings due to numerous possible confounding factors that are eliminated in the experimental design. Future highly standardized in vitro experiments and clinical studies should focus on the influence of different guiding concepts and types of partial edentulism on the accuracy of sCAIS.

## Figures and Tables

**Table 1:** Descriptive statistics of each of the variables: guiding system and site of implantation. DK—drill key, KL—keyless, AntExt—anterior extended edentulous area, AntSTG—anterior single-tooth gap, ProPosSTG—proximal posterior single-tooth gap, DisPosSTG—distal posterior single-tooth gap, ProDE—proximal site of distal extension, DisDE—distal site of distal extension.

Guiding System	Site of Implantation	Min	Q1	Median	Q3	Max	Mean	SD
3D Deviation at crest (mm)								
DK	AntExt	0.11	0.32	0.37	0.51	0.79	0.41	0.17
DK	AntSTG	0.47	0.66	0.83	0.87	0.91	0.77	0.14
DK	ProPosSTG	0.53	0.58	0.72	0.79	0.90	0.70	0.13
DK	DisPosSTG	0.70	0.99	1.10	1.13	1.15	1.03	0.16
DK	ProDE	0.64	0.72	0.86	0.98	1.23	0.87	0.18
DK	DisDE	0.51	0.62	0.77	0.89	0.94	0.75	0.15
I KL	AntExt	0.43	0.64	0.89	1.03	1.29	0.84	0.26
KL	AntSTG	0.50	0.74	0.95	1.26	1.39	0.96	0.29
KL	ProPosSTG	0.54	0.80	0.97	1.15	1.33	0.96	0.26
KL	DisPosSTG	0.52	0.68	0.84	0.95	1.02	0.80	0.17
KL	ProDE	0.42	0.57	0.77	0.86	0.92	0.73	0.17
KL	DisDE	0.40	0.51	0.66	0.98	1.32	0.75	0.30
DK	Overall	0.11	0.52	0.72	0.89	1.23	0.71	0.26
KL	Overall	0.40	0.63	0.86	0.98	1.39	0.84	0.26
3D Deviation at apex (mm)								
DK	AntExt	0.25	0.39	0.58	0.68	1.19	0.59	0.16
DK	AntSTG	0.61	0.79	1.13	1.26	1.43	1.07	0.27
DK	ProPosSTG	0.80	0.97	1.12	1.52	1.70	1.21	0.31
DK	DisPosSTG	1.14	1.29	1.62	1.74	2.05	1.57	0.28
DK	ProDE	0.70	0.80	1.09	1.22	1.51	1.05	0.26
DK	DisDE	0.95	1.19	1.55	1.67	2.05	1.49	0.32
KL	AntExt	0.65	1.24	1.54	1.86	2.17	1.50	0.43
KL	AntSTG	0.78	1.13	1.32	1.44	1.55	1.27	0.23
KL	ProPosSTG	0.76	1.11	1.60	1.90	2.36	1.56	0.48
KL	DisPosSTG	0.72	0.85	1.05	1.22	1.30	1.03	0.21
KL	ProDE	0.49	0.82	1.03	1.30	1.50	1.04	0.31
KL	DisDE	0.41	0.92	1.05	1.34	1.51	1.06	0.31
DK	Overall	0.25	0.70	1.13	1.45	2.05	1.08	0.45
KL	Overall	0.41	0.98	1.26	1.52	2.36	1.28	0.41
Angular deviation (°)								
DK	AntExt	0.24	1.12	1.52	2.11	3.47	1.02	0.73
DK	AntSTG	1.19	2.13	2.63	3.41	4.15	2.69	0.89
DK	ProPosSTG	1.96	2.59	3.52	4.96	5.20	3.56	1.18
DK	DisPosSTG	4.63	5.03	6.06	6.49	7.78	5.92	0.98
DK	ProDE	0.54	1.37	1.79	2.60	4.11	2.02	1.06
DK	DisDE	2.34	3.09	4.23	4.75	5.46	3.99	1.04
KL	AntExt	1.72	3.70	4.33	5.44	5.88	4.37	1.10
KL	AntSTG	1.29	2.25	2.72	3.18	3.58	2.65	0.67
KL	ProPosSTG	1.45	2.91	4.68	5.44	7.02	4.26	1.71
KL	DisPosSTG	1.55	1.88	2.36	3.30	4.28	2.56	0.87
KL	ProDE	1.56	1.81	2.64	4.22	5.43	2.97	1.32
KL	DisDE	0.36	1.59	2.63	3.41	4.16	2.43	1.24
DK	Overall	0.24	1.69	2.58	4.33	7.78	3.06	1.72
KL	Overall	0.36	2.25	3.22	4.41	7.02	3.37	1.43
Vertical linear deviation (mm)								
DK	AntExt	0.00	0.06	0.17	0.34	0.49	0.20	0.16
DK	AntSTG	0.40	0.55	0.62	0.73	0.82	0.62	0.12
DK	ProPosSTG	0.33	0.37	0.42	0.55	0.76	0.46	0.13
DK	DisPosSTG	0.67	0.89	1.01	1.04	1.07	0.95	0.15
DK	ProDE	0.33	0.50	0.65	0.80	0.94	0.64	0.20
DK	DisDE	0.00	0.01	0.05	0.08	0.13	0.05	0.04
KL	AntExt	0.00	0.18	0.29	0.39	0.57	0.29	0.15
KL	AntSTG	0.34	0.49	0.55	1.00	1.18	0.67	0.29
KL	ProPosSTG	0.08	0.38	0.53	0.63	0.75	0.48	0.22
KL	DisPosSTG	0.49	0.61	0.73	0.82	0.89	0.71	0.14
KL	ProDE	0.08	0.12	0.20	0.60	0.78	0.33	0.27
KL	DisDE	0.02	0.17	0.33	0.62	1.32	0.43	0.38
DK	Overall	0.00	0.13	0.45	0.67	1.07	0.45	0.33
KL	Overall	0.00	0.24	0.46	0.62	1.32	0.46	0.29

## Data Availability

The raw data supporting the conclusions of this article will be made available by the authors on request.

## Supplementary Materials

The following supporting information can be downloaded at: https://www.mdpi.com/article/10.3390/medicina61040617/s1, Table S1: Summary of the Kruskal–Wallis test and post hoc pairwise comparison analysis on differences between deviations from planned implant positions in subgroups of implantation sites.

## Abbreviations

The following abbreviations are used in this manuscript:

3D	Three Dimensional
CBCT	Cone Beam Computed Tomography
DLP	Digital Light Processing
FDI	Federal Dentaire Internationale
sCAIS	Static Computer-Assisted Implant Surgery
SLS	Selective Laser Sintering
STG	Single-tooth gap
STL	Standard Tessellation Language
