# Supplementary material for: The Influence of Guiding Concept on the Accuracy of Static Computer-Assisted Implant Surgery in Partially Edentulous Cases: An In Vitro Study
Source: Medicina (Kaunas). 2025 Mar 28;61(4):617. doi: 10.3390/medicina61040617 (PMC12028878; doi:10.3390/medicina61040617)
Supplement: Supplementary file 1 [file medicina-61-00617-s001.zip › medicina-3536191-supplementary.pdf]

Outcomes	Kruskal-Wallis H test	***P* value (post hoc)**														
		AntExt-AntSTG	AntExt-ProPosSTG	AntExt-DisPosSTG	AntExt-ProDE	AntExt-DisDE	AntSTG-ProPosSTG	AntSTG-DisPosSTG	AntSTG-ProDE	AntSTG-DisDE	ProPosSTG-DisPosSTG	ProPosSTG-ProDE	ProPosSTG-DisDE	DisPosSTG-ProDE	DisPosSTG-DisDE	ProDE-DisDE
**Keyless**																
**3D crestal deviation**	p <0 .001* $\chi2(5)$ = 43.553	< 0.001[a]	0.008	< 0.001[a]	< 0.001[a]	< 0.001[a]	0.472	0.035	0.404	0.921	0.005	0.120	0.535	0.202	0.027	0.350
**3D apical deviation**	p < 0.001* $\chi2(5)$ = 44.498	0.006	< 0.001[a]	< 0.001[a]	0.008	< 0.001[a]	0.510	0.011	0.934	0.040	0.061	0.458	0.164	0.009	0.629	0.033
**Angular deviation**	p < 0.001* $\chi2(5)$ = 46.557	0.034	< 0.001[a]	< 0.001[a]	0.468	< 0.001[a]	0.240	< 0.001[a]	0.229	0.088	0.031	0.017	0.594	< 0.001[a]	0.105	0.004
**Vertical linear**	p < 0.001* $\chi2(5)$ = 54.998	< 0.001[a]	0.037	< 0.001[a]	< 0.001[a]	0.152	0.204	0.094	0.991	< 0.001[a]	0.003	0.200	0.002[a]	0.096	< 0.001[a]	< 0.001[a]
**Drill - key**																
**3D crestal deviation**	p = 0.161 $\chi2(5)$ = 7.916	0.351	0.240	0.689	0.191	0.267	0.835	0.249	0.052	0.077	0.173	0.032	0.048	0.432	0.538	0.865
**3D apical deviation**	p = 0.002* $\chi2(5)$ = 19.376	0.266	0.842	0.002[a]	0.005	0.01	0.255	0.083	0.139	0.202	0.004	0.009	0.016	0.796	0.644	0.839
**Angular deviation**	p < 0.001* $\chi2(5)$ = 24.306	0.001[a]	0.590	< 0.001[a]	0.006	< 0.001[a]	0.022	0.809	0.713	0.856	0.011	0.054	0.013	0.542	0.952	0.583
**Vertical linear**	p < 0.001* $\chi2(5)$ = 25.354	< 0.001[a]	0.039	< 0.001[a]	0.753	0.302	0.281	0.448	0.009	0.049	0.066	0.129	0.370	< 0.001[a]	0.006	0.535

*Statistically significant in Kruskal-Wallis test (*P* < .05).
[a] Statistically significant in post hoc after Kruskal-Wallis under Dunn's test with p < .003 accepted as significant after Bonferroni correction.

Table S1. Summary of the Kruskal-Wallis test and post hoc pairwise comparison analysis on differences between deviations from planned implant positions in subgroups of implantation sites.
